# Group V Secreted Phospholipase A_2_ Induces the Release of Proangiogenic and Antiangiogenic Factors by Human Neutrophils

**DOI:** 10.3389/fimmu.2017.00443

**Published:** 2017-04-19

**Authors:** Stefania Loffredo, Francesco Borriello, Raffaella Iannone, Anne L. Ferrara, Maria R. Galdiero, Vincenzo Gigantino, Pasquale Esposito, Gilda Varricchi, Gerard Lambeau, Marco A. Cassatella, Francescopaolo Granata, Gianni Marone

**Affiliations:** ^1^Division of Clinical Immunology and Allergy, Center for Basic and Clinical Immunology Research (CISI), University of Naples Federico II, Naples, Italy; ^2^Division of Infectious Diseases, Department of Medicine, Boston Children’s Hospital, Boston, MA, USA; ^3^Pathology Unit, Istituto Nazionale Tumori Fondazione “G. Pascale”, Naples, Italy; ^4^U.O.C. Immunohematology and Transfusion Medicine, University of Naples Federico II, Naples, Italy; ^5^CNRS, Institut de Pharmacologie Moléculaire et Cellulaire, Université Côte d’Azur, Valbonne Sophia Antipolis, France; ^6^Department of Medicine, Division of General Pathology, University of Verona, Verona, Italy; ^7^CNR Institute of Experimental Endocrinology and Oncology “G. Salvatore”, Naples, Italy

**Keywords:** secreted phospholipase A_2_, neutrophil, vascular endothelial growth factor, angiopoietin, lung tumor, integrin, PLA_2_R1

## Abstract

Secreted phospholipases A_2_ (sPLA_2_s) are extracellular enzymes that catalyze the release of free fatty acids and lysophospholipids from membrane phospholipids and also bind to different receptors (e.g., PLA_2_R1 or integrins). To date, 12 mammalian sPLA_2_s have been identified, which play a critical role in pathophysiological processes including inflammation and cancer. sPLA_2_s activate immune cells such as human neutrophils (PMNs) by enzymatic activity- or receptor-mediated mechanisms. In addition, human PMNs synthesize and store human group V (hGV) and human group X (hGX) sPLA_2_s in their granules, but only the former is released upon cellular activation. We investigated the effects of sPLA_2_s on the release of proangiogenic and antiangiogenic factors by PMNs. We found that exogenous hGV and hGX sPLA_2_s induce the release of vascular endothelial growth factor (VEGF)-A, angiopoietin 1 (Ang1), and CXCL8/IL-8. Only hGV induces the secretion of the antiangiogenic isoform of VEGF-A, namely, VEGF-A_165b_. While the release of VEGF-A, Ang1, and CXCL8/IL-8 was likely mediated by hGV enzymatic activity and/or binding to PLA_2_R1 and heparan sulfate proteoglycans, the release of VEGF-A_165b_ requires the interaction with α_V_β_3_ and α_4_β_1_ integrins. We also provide evidence that endogenous hGV released by *N*-formyl-met-leu-phe (fMLF)-activated PMNs is involved in the release of angiogenic factors. The translational relevance of these data is supported by our findings that hGV expression is increased in human samples of lung cancer which are infiltrated by PMNs. Overall, our results suggest that the hGV–neutrophil axis may play a relevant role in the modulation of cancer-related inflammation and angiogenesis.

## Introduction

Secreted phospholipases A_2_ (sPLA_2_s) are extracellular enzymes that catalyze the hydrolysis of the *sn* − 2 position of membrane glycerophospholipids to release free fatty acids and lysophospholipids, thereby regulating several processes including the production of lipid mediators (1). However, sPLA_2_ effects are not related only to their enzymatic activity but also to their ability to activate target cells through the engagement of different targets [e.g., PLA_2_R1, heparan sulfate proteoglycans (HSPGs), integrins] (2–9). To date, 12 mammalian sPLA_2_s, namely, groups IB, IIA, IIC, IID, IIE, IIF, III, V, X, XIIA, XIIB, and otoconin-95, have been identified (5, 10). They often play critical roles in pathophysiological processes, including inflammation and cancer (1, 5, 11, 12). Indeed, sPLA_2_s are expressed in inflamed tissues and tumors (1, 12–15). In addition, several immune cells are both sources and/or targets of sPLA_2_s (16–21). In particular, sPLA_2_s activate human neutrophils (PMNs) inducing elastase and CXCL8/IL-8 release and activating ERK1/2 and p38 MAP kinases by a receptor-mediated mechanism (8, 21–23). In addition, PMNs store human group V (hGV) and human group X (hGX) in their granules (16, 19), but only the former is released in response to the bacterial *N*-formylmethionyl peptide, formyl-methionyl-leucylphenylalanine (fMLF) (16, 19).

PMNs are innate immune cells with primary roles in the acute phase of inflammation and resistance against invading pathogens (24). Because of their terminally differentiated phenotype and short half-life, the role of PMNs in tumor development has been considered marginal. Recent evidence changed this point of view. Indeed, tumor-associated neutrophils (TAN) can exert anti-tumoral as well as pro-tumoral functions and findings derived from murine models suggest that PMNs display unsuspected plasticity (25, 26). Epidemiological studies indicate an association between TAN and clinical outcome in several but not all tumors (27–33). In early stages of lung tumors, PMNs exert immunostimulatory properties but acquire immunosuppressive features as the disease progresses (34, 35). There is some evidence that PMNs can modulate tumor initiation and growth through the production of angiogenic factors (36), but further investigations are required to better understand the role of PMNs in modulating tumor angiogenesis.

Angiogenesis, the formation of new blood vessels, and lymphangiogenesis, the formation of new lymphatic vessels, are complex processes that require the coordinated action of several factors, namely, vascular endothelial growth factors (VEGFs) and angiopoietins [angiopoietin 1 (Ang1) and Ang2] (37, 38). Several proangiogenic and antiangiogenic factors have been identified. VEGF-A and VEGF-B are key mitogens for endothelial cells and can act both as pro- and antiangiogenic factors due to the different spliced forms (39–41). For instance, the splicing variant VEGF-A_165_ exists in two different isoforms: VEGF-A_165a_ is the most potent proangiogenic variant, whereas VEGF-A_165b_ is an antiangiogenic isoform (42–44). Endothelial cell maturation is also promoted by the angiopoietins (Ang1 and Ang2), whose role can be either proangiogenic or antiangiogenic depending on the microenvironment (45, 46). The key regulators of lymphangiogenesis are VEGF-C and VEGF-D (38, 47).

Immune cells are important sources as well as targets of proangiogenic and antiangiogenic factors (48). In particular, PMNs release a variety of proangiogenic and antiangiogenic factors and play important roles in several models of inflammatory and tumor angiogenesis (36, 49–51). Indeed, PMNs release VEGF-A in response to fMLF, LPS, and phorbolmyristate acetate (PMA), while Ang1 is secreted only in response to PMA (50). It is unknown whether sPLA_2_s can modulate the production of proangiogenic and antiangiogenic factors from PMNs.

Since we demonstrated that sPLA_2_s induce the production of angiogenic factors from human macrophages (17), in this study, we sought to investigate the production of pro- and antiangiogenic factors by PMNs in response to different forms of sPLA_2_s.

## Materials and Methods

### Reagents

The following were purchased: l-glutamine, antibiotic–antimycotic solution (10,000 IU/ml penicillin, 10 mg/ml streptomycin, and 25 µg/ml amphotericin B), Triton X-100, Histopaque^®^-1077, bovin serum albumin (BSA), Heparinase I and III Blend from *Flavobacterium heparinum, N*-formyl-met-leu-phe (fMLF), phenolphthalein β-d-glucuronide sodium salt, detoxified LPS (from *E. coli* serotype 0111:B4), PMA, brefeldin A, and cycloheximide (Sigma-Aldrich, St. Louis, MO, USA); RPMI and fetal calf serum (FCS, endotoxin level <0.1 EU/ml) (MP Biomedicals Europe, Illkirch, France); P11, TCS 2314 (Tocris Bioscience, UK); anti-human VEGF-A_165_ (monoclonal mouse IgG_2B_; Clone 26603) and anti-human VEGF_165b_ (monoclonal mouse IgG_1_; Clone 56-1) (R&D System, Minneapolis, MN, USA). Target-specific primers for *VEGFA_165a_, VEGFA_165b_, VEGFB, VEGFC, VEGFD, Ang1, Ang2*, and β*-actin* were produced and purified by Custom Primers (Life Technologies, Milan, Italy). The recombinant sPLA_2_s human group IB, hGIIA, hGIIE, hGIIF, hGV, hGX, and hGXIIA and the inhibitors Me-Indoxam and RO092906A were prepared in the laboratory of Gerard Lambeau and were a generous gift from and Michael H. Gelb (Departments of Chemistry and Biochemistry, University of Washington, Seattle, WA, USA). sPLA_2_ preparations were routinely checked for LPS contamination (*Limulus amebocyte* Test, MP Biomedicals) and discarded if the LPS concentration was above the detection limit of the assay (0.125 EU/ml). All other reagents were from Carlo Erba (Milan, Italy).

### Isolation and Purification of Human Neutrophils

Granulocytes were isolated from buffy coats of healthy donors obtained from the Leukapheresis Unit. After dextran sedimentation, PMNs were obtained by centrifugation over Histopaque^®^-1077 at 400 × *g* for 30 min, at 22°C, at a 1:1 ratio. Finally, PMNs were isolated by negatively removing all contaminating cells using the MACSxpress Neutrophil Isolation Kit and MACSxpress Erythrocyte Depletion Kit (Miltenyi Biotec, Bologna, Italy). This procedure yields a population of CD66b^+^ cells with a purity greater than 99% as assessed by flow cytometry. PMNs were suspended (5 × 10^6^ cells/ml) in complete medium (RPMI 1640 containing 5% FCS, 2 mM l-glutamine, and 1% antibiotic–antimycotic solution) and incubated in different plates (Falcon, Becton Dickinson, Franklin Lakes, NJ, USA) at 37°C in a humidified atmosphere of 5% CO_2_ and 95% air. After 30 min of rest, the cells were used for the experiments.

### Cell Incubations

PMNs were incubated (37°C, 5 min–3 h) in RPMI 1640 containing 5% FCS, 2 mM l-glutamine, and 1% antibiotic–antimycotic solution and stimulated with various concentrations (0.3–10 µg/ml) of human GIB, GIIA, GIIE, GIIF, GIII, GV, GX, GXIIA, fMLF (50 nM), detoxified LPS (100 ng/ml), and PMA (80 nM). In selected experiments, hGV and hGX (3 µg/ml) were preincubated (37°C, 20 min) with increasing concentrations (0.01–10 µM) of their inhibitors Me-Indoxam or RO092906A. In other experiments, PMNs were preincubated (37°C, 1 h) with heparinase (0.4 U/ml) or (37°C, 30 min) with P11 (100 nM) and/or TCS 2314 (100 nM), brefeldin A (10 µg/ml), cycloheximide (10 µg/ml) and then stimulated (37°C, 30 min) with hGV (3 µg/ml). In selected experiments, PMNs were preincubated (37°C, 15 min) with Me-Indoxam or RO092906A and then stimulated with fMLF (37°C, 10 min). At the end of the experiment, the supernatants were removed, centrifuged (1,000 × *g*, 4°C, 5 min) and stored at −80°C for subsequent determination of mediator release.

### RT-PCR

Total cellular RNA was isolated from PMNs using the SV RNA isolation system (Promega, Madison, WI, USA), treated with RNase-free DNase I and resuspended in DEPC water. RNA concentration and quality were assessed by spectrophotometry. Total mRNA was reverse-transcribed (Superscript III Reverse Transcriptase 200 U, Life Technologies) and quantitative PCR (qPCR) was carried out in iCycler-iQ5 real-time PCR detection system (Bio-Rad, Hercules, CA, USA) using SYBR Green Master Mix (Bio-Rad). Target-specific primers for *VEGF_165a_, VEGFA_165b_, VEGFB, VEGFC, VEGFD, Ang1, Ang2*, and β*-actin* suitable for qPCR were produced and purified by Custom Primers (Life Technologies, Milan, Italy) and are reported in Table 1. β*-Actin* was used as housekeeping gene to normalize cycle threshold (*Ct*) values using the 2^−Δ^*^Ct^* formula. The data were analyzed with iCycler iQ analysis software (Bio-Rad).

**Table 1:** **Primer sequences and conditions for quantitative PCR**.

Target	Product length (bp)	Ta (°C)	Primer (5′–3′)	GenBank accession no. or reference
VEGFA_165a_	79	60	Forward: GCCTTGCCTTGCTGCTCTAC	NM_003376
			Reverse: TGATTCTGCCCTCCTCCTTCTG	
VEGFA_165b_	157	60	Forward: GAGCAAGACAAGAAAATCCC	(43)
			Reverse: GTGAGAGATCTGCAAGTACG	
VEGFB	128	60	Forward: AGGACAGAGTTGGAAGAGGAG	NM-003377
			Reverse: AGGAAGAGCCAGTTGTAAGATG	
VEGFC	197	60	Forward: ATGTTTTCCTCGGATGCTGGA	NM_005429
			Reverse: CATTGGCTGGGGAAGAGTTT	
VEGFD	226	60	Forward: GTATGGACTCTCGCTCAGCAT	NM_004469
			Reverse: AGGCTCTCTTCATTGCAACAG	
Ang1	73	60	Forward: CAGGAGGATGGTGGTTTGATG	NM_001314051
			Reverse: TGGTTTTGTCCCGCAGTATAGAA	
Ang2	65	60	Forward: TTCCTCCTGCCAGAGATGGA	NM_001118888
			Reverse: TGCACAGCATTGGACACGTA	
β-Actin	99	60	Forward: TGCGTGACATTAAGGAGAAG	NM_001101
			Reverse: GCTCGTAGCTCTTCTCCA	

*Ta, annealing temperature*.

### Mediator Release Assays

The concentration of VEGF-A, VEGFA_165b_, VEGF-B, VEGF-C, VEGF-D, Ang1, Ang2, and CXCL8/IL-8 in the supernatants, lysed or freshly isolated PMNs (lysed in Tryton 0.1%) was measured in duplicate determinations using commercially available ELISA kits (R&D System). The ELISA sensitivity is 31.1–2,000 pg/ml for VEGF-A, 31.1–4,000 pg/ml for VEGF-A_165b_, 9.4–300 pg/ml for VEGF-B, 62–4,000 pg/ml for VEGF-C, 31.1–2,000 pg/ml for VEGF-D, 156.25–10,000 pg/ml for Ang1, 31.1–4,000 pg/ml for Ang2, and 31.1–2,000 pg/ml for CXCL8/IL-8. β-Glucuronidase release was measured with a colorimetric assay.

### PLA_2_ Activity Assay

A modified liposomal-based fluorescent assay was used to measure PLA_2_ activity in neutrophil supernatants. Briefly, a PLA_2_ substrate cocktail consisting of 7-hydroxycoumarinyl-arachidonate (0.3 mM), 7-hydroxycoumarinyl-linolenate (0.3 mM), hydroxycoumarinyl-6 heptenoate (0.3 mM), 10 mM dioleoylphosphatidylcholine (DOPC), and 10 mM dioleoylphosphatidylglycerol (DOPG) was prepared in ethanol. Liposomes were formed by gradually adding 77 µl substrate/lipid cocktail to 10 ml PLA_2_ buffer (50 mM Tris–HCl at pH 8.9, 100 mM NaCl, 1 mM CaCl_2_) while stirring rapidly over 1 min using a magnetic stirrer (Invitrogen EnzChek^®^ phospholipase A_2_ assay). Neutrophil supernatants (50 µl) was added to 96-well plates, and PLA_2_ activity was initiated by adding 50 µl substrate cocktail. Fluorescence (excitation at 360 nm and emission at 460 nm) was measured, and specific activity (relative fluorescent units/μg protein/min) for each sample was calculated.

### Immunohistochemistry Analysis of Non-Tumor and Tumor Lung Tissues for hGV and CD66b

Non-tumor and tumor lung tissues were obtained from patients affected by lung adenocarcinoma (HCV^−^, HBsAg^−^, and HIV-1^−^) undergoing lung resection. Immunohistochemical staining has been carried out on 4-µm lung cancer serial sections from formalin-fixed, paraffin-embedded tissues, in order to evaluate the expression of hGV and CD66b. Negative control slides without primary antibody were included for each staining (not shown). Paraffin slides were deparaffinized in xylene and rehydrated through graded alcohols. Antigen retrieval was performed with slides heated in 1 mM EDTA buffer (pH 9.0) for hGV and 0.01 M citrate buffer (pH 6.0) for CD66b, in a bath for 20 min at 97°C. After antigen retrieval, the slides were allowed to cool and rinsed with TBS. The endogenous peroxidase activity was inactivated with 3% hydrogen peroxide. Following the protein block (BSA 5% in PBS 1×), the slides were incubated with a polyclonal rabbit antibody against hGV (NBP2-31558, Novus Biologicals, Littleton, CO, USA, dilution 1:100) or a monoclonal mouse anti-human CD66b antibody (555723, BD Pharmingen, San Jose, CA, USA, dilution 1:400 at 4°C overnight). The sections were rinsed in TBS and incubated with biotinylated anti-rabbit or anti-mouse antibodies, respectively, for 1 h at room temperature. Immunoreactivity was visualized using 3,3′-diaminobenzidine (DAB) and avidin–biotin–peroxidase complex. Finally, sections were weakly counterstained with hematoxylin, mounted, and interpreted using light microscope.

### Statistical Analysis

The data are expressed as mean values ±SD of the indicated number of experiments. Statistical analysis was performed with Prism 6 (GraphPad Software) by one-way analysis of variance followed by Dunnett’s test (when comparison was made against a control) or Bonferroni’s test (when comparison was made between each pair of groups). Statistically significant differences were accepted when the *p* value was at least ≤0.05.

## Results

### sPLA_2_s Induce the Release of Proangiogenic and Antiangiogenic Factors by PMNs

**Figure 1:**
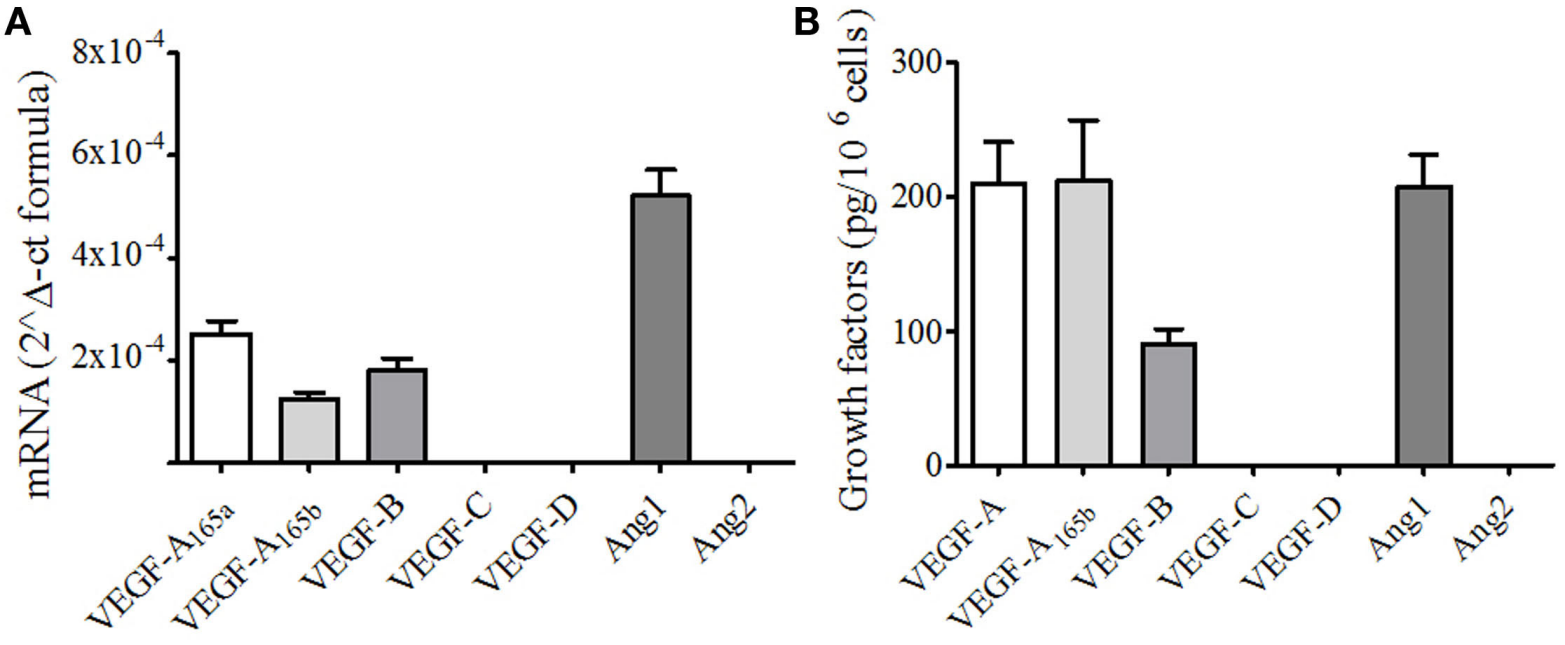
**Human neutrophils (PMNs) constitutively express different forms of vascular endothelial growth factors (VEGF) and angiopoietins**. **(A)** *VEGFA*_165a_, *VEGFA*_165b_, *VEGFB, VEGFC, VEGFD, Ang1*, and *Ang2* mRNA expression in PMNs. The results are the mean ± SD of four different preparations of PMNs. RNA extraction from resting PMNs and RT-PCR was performed as described under Section “Materials and Methods.” **(B)** Detection of VEGF and Ang proteins. Freshly isolated PMNs were lysed in Tryton 0.1%, and the concentrations of VEGFs and Angs were determined by ELISA. The results are the mean ± SD of six different preparations of PMNs.

To evaluate the impact of sPLA_2_ on the production of angiogenic and lymphangiogenic factors by human PMNs, we first assessed the basal expression of VEGFs and Angs. Freshly isolated PMNs constitutively expressed VEGF-A, VEGF-B, and Ang1 at both mRNA and protein levels. By contrast, no expression of VEGF-C and VEGF-D (lymphangiogenic factors) and Ang2 could be detected (Figures 1A,B). Interestingly, PMNs constitutively expressed VEGF-A_165b_ (Figures 1A,B), the antiangiogenic splice variant of VEGF-A_165_ (42).

**Figure 2:**
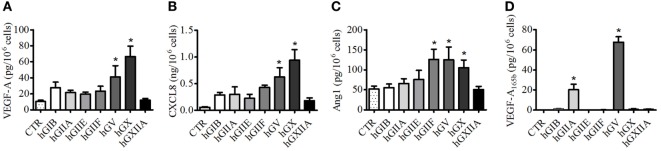
**Human secreted phospholipases A_2_ (sPLA_2_s) induce the release of vascular endothelial growth factors (VEGFs), Ang1, and CXCL8/IL-8 from PMNs**. PMNs were incubated (37°C, 3 h) with sPLA_2_ [5 µg/ml, human group IB (hGIB), hGIIA, hGIIE, hGIIF, hGV, hGX, and hGXIIA] or control medium **(A–D)**. At the end of incubation, the supernatants were collected and centrifuged (1,000 × *g*, 4°C, 5 min). VEGF-A **(A)**, CXCL8/IL-8 **(B)**, Ang1 **(C)**, and VEGF-A_165b_ **(D)** were determined by ELISA. The values are expressed as picograms or nanograms of mediators per 10^6^ cells. The results are the mean ± SD of eight different preparations of PMNs. *p < 0.05 vs. control.

We then evaluated the effects of several human recombinant sPLA_2_s on the secretion of VEGF-A, VEGF-A_165b_, VEGF-B, and Ang1 as well as the proangiogenic chemokine CXCL8/IL-8 from PMNs. Several sPLA_2_s induced the release of VEGF-A (Figure 2A) and CXCL8/IL-8 (Figure 2B) and promoted the release of Ang1 (Figure 2C). However, at the concentration used (5 µg/ml), the release of VEGFA and CXCL8 was significant upon stimulation with hGV and hGX, and the release of Ang1 was significant upon stimulation with hGIIF, hGV, and hGX. These results were paralleled by the highest β-glucuronidase release used as a marker of exocytosis (Figure S1A in Supplementary Material) suggesting the release of preformed mediators rather than *de novo* synthesis. By contrast, no secretion of VEGF-B (Figure S1B in Supplementary Material) could be observed in any of the tested conditions. Interestingly, hGV and, to a lesser extent, hGIIA were the only sPLA_2_s to induce the secretion VEGF-A_165b_ (Figure 2D).

**Figure 3:**
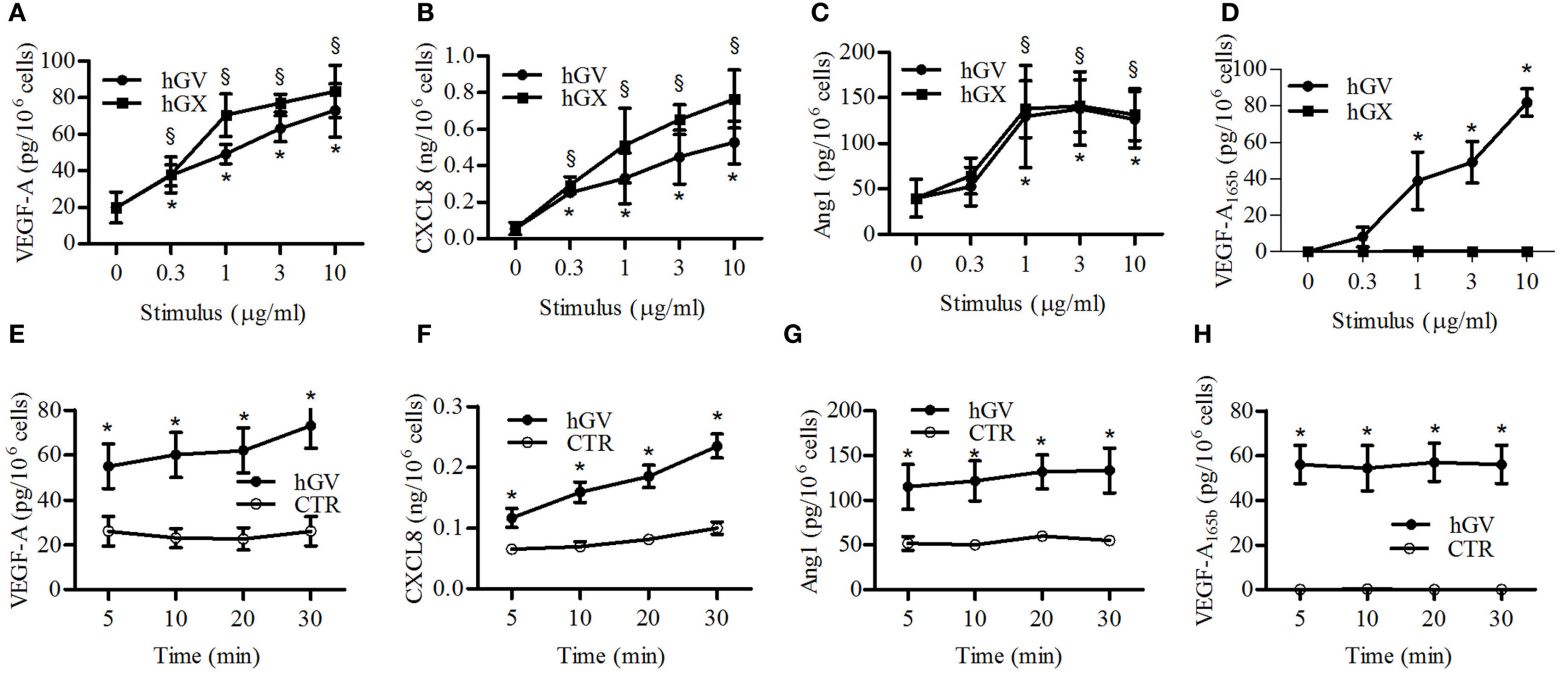
**Effect of increasing concentrations of human group V (hGV) and human group X (hGX) on vascular endothelial growth factor (VEGF)-A (A), CXCL8/IL-8 (B), angiopoietin 1 (Ang1) (C), and VEGF-A_165b_ (D) release from PMNs**. PMNs were incubated (37°C, 3 h) with hGV and hGX (0.3–10 µg/ml) or control medium. **(E–H)** Kinetics of hGV-induced release of VEGFs, Ang1, and CXCL8/IL-8 from PMNs. The cells were incubated (37°C, 5–30 min) with hGV (3 µg/ml). At the end of incubations, the supernatants were collected and centrifuged (1,000 × *g*, 4°C, 5 min). Data are the mean ± SD of different eight preparations of PMNs *(for hGV) and ^§^(for hGX) *p* < 0.05 vs. control.

To better understand the mechanisms of sPLA_2_ neutrophil stimulation, we performed dose–response and time-dependent experiments with hGV and hGX sPLA_2_s, which are more effective in stimulating human PMNs and are the only sPLA_2_s expressed by these cells (16). Figure 3 shows that the release of VEGF-A (Figure 3A), CXCL8/IL-8 (Figure 3B), and Ang1 (Figure 3C) in response to hGV and hGX was induced by concentrations as low as 1 µg/ml. By contrast, Figure 3D shows that VEGF-A_165b_ secretion was observed only with hGV, but not with hGX, at all tested concentrations. Moreover, hGV induced the release of these mediators as early as after 5 min of stimulation (Figures 3E,H).

To verify whether hGV-induced secretion of proangiogenic and antiangiogenic mediators requires *de novo* protein synthesis, neutrophils were stimulated with hGV in the presence or absence of brefeldin A (an inhibitor of anterograde cellular transport and protein secretion) or cycloheximide (an inhibitor of protein synthesis). Neither brefeldin A nor cycloheximide affected the spontaneous release of VEGF-A, CXCL8/IL-8, Ang1, and VEGF-A_165b_ (data not shown). However, brefeldin A but not cycloheximide significantly inhibited the release of these mediators induced by hGV (Table S1 in Supplementary Material). Moreover, we measured VEGF-A, Ang1, and VEGF-A_165b_ protein levels in supernatants and cellular lysates of unstimulated and hGV-stimulated PMNs and found that total protein levels (supernatants plus cellular lysates) of angiogenic mediators were not significantly modulated by hGV stimulation (Table S2 in Supplementary Material).

**Figure 4:**
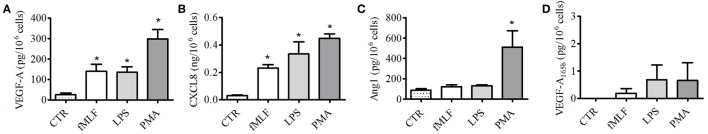
**Effect of fMLF, LPS, and phorbolmyristate acetate (PMA) on vascular endothelial growth factors (VEGFs), angiopoietin 1 (Ang1), and CXCL8/IL-8 release from PMNs**. PMNs were incubated (37°C, 3 h) with fMLF (50 nM), LPS (100 ng/ml), PMA (80 nM), or control medium **(A–D)**. At the end of incubation, the supernatants were collected and centrifuged (1,000 × *g*, 4°C, 5 min). VEGF-A **(A)**, CXCL8/IL-8 **(B)**, Ang1 **(C)**, and VEGF-A_165b_ **(D)** were determined by ELISA. The values are expressed as picograms or nanograms of mediators per 10^6^ cells. The results are the mean ± SD of eight different preparations of PMNs. *p < 0.05 vs. control.

To corroborate these findings and to exclude a non-specific PMN activation by sPLA_2_, we assessed the secretion of VEGF-A, VEGF-A_165b_, Ang1, and CXCL8/IL-8 following the stimulation of PMNs with well-known neutrophil stimuli, such as fMLF, PMA, and LPS. Even though all these stimuli induced the release of VEGF-A (Figure 4A) and CXCL8/IL-8 (Figure 4B), the secretion of Ang1 (Figure 4C) was only induced by PMA. By contrast, none of the tested stimuli induced the release of VEGF-A_165b_ (Figure 4D). Taken together, these results indicate that hGV was the only sPLA_2_ able to induce the release of preformed pro- and antiangiogenic molecules, whereas the effect of hGX was limited to proangiogenic factors.

### hGV-Induced Secretion of Angiogenic and Antiangiogenic Factors Requires the Interaction with Different Targets

**Figure 5:**
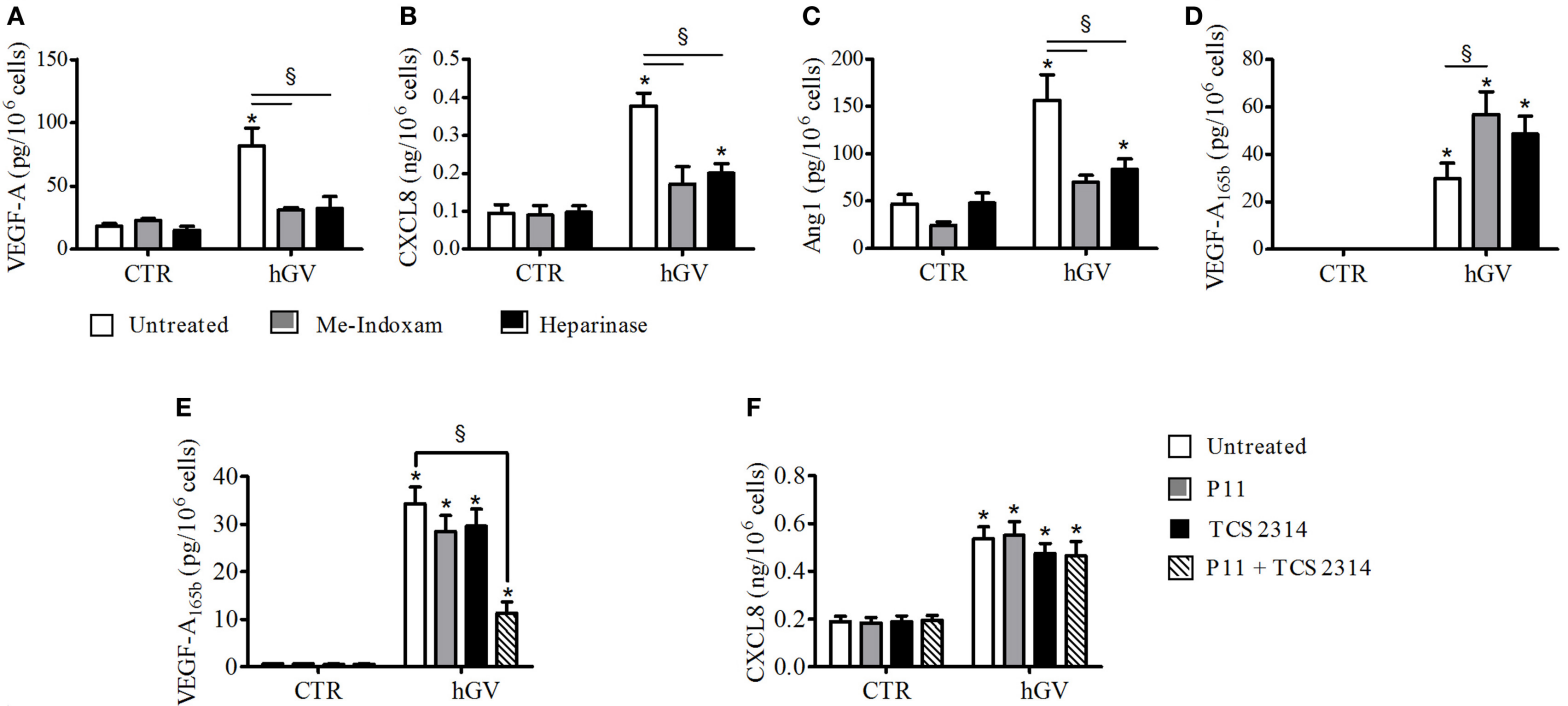
**(A–D)** Effect of Me-Indoxam or with Heparinase on human group V (hGV)-induced vascular endothelial growth factor (VEGF)-A, CXCL8/IL-8, Ang1, and VEGF-A_165b_ release from PMNs. hGV (3 µg/ml) was preincubated (37°C, 20 min) with Me-Indoxam (0.1 µM) or control medium. PMNs were then incubated (37°C, 1 h) with heparinase (0.4 U/ml) or control medium and then stimulated (37°C, 30 min) with hGV alone or with the combination of hGV with Me-Indoxam. **(E,F)** α_V_β_3_ (P11) and α_4_β_1_ (TCS2314) receptor antagonists inhibit GV-induced PMNs production of antiangiogenic factors. PMNs were preincubated (37°C, 30 min) with or without P11 and TCS 2314 (100 nM) and then stimulated (37°C, 30 min) with hGV (3 µg/ml). Data are the mean ± SD of eight different preparations of PMNs. *p < 0.05 vs. respective control. ^§^*p* < 0.05 vs. hGV alone.

Several evidences demonstrate that PMNs express PLA_2_R1 that is involved in sPLA_2_-induced neutrophil activation (8, 21–23). To verify whether hGV enzymatic activity and/or PLA_2_R1 were involved in the production of pro- and antiangiogenic factors induced by hGV, we stimulated PMNs in the presence of Me-Indoxam. This inhibitor protrudes out of the catalytic groove when bound to sPLA_2_s, thereby leading to steric hindrance and hence interfering with the sPLA_2_–receptor interaction (52). We have previously shown that Me-Indoxam prevents receptor-mediated activation of HLMs and PMNs stimulated with sPLA_2_s (17, 18, 21, 53). hGV also binds HSPGs that mediate its internalization (8). To verify a possible role for HSPGs, PMNs were pre-treated with heparinase to eliminate surface HSPGs before stimulation with hGV. Both Me-indoxam and heparinase pre-treatment markedly reduced the secretion of VEGF-A (Figure 5A), CXCL8/IL-8 (Figure 5B), and Ang1 (Figure 5C). Surprisingly, the release of VEGF-A_165b_ (Figure 5D) was not inhibited, instead it was significantly increased by Me-Indoxam and to a lesser extent also by heparinase pre-treatment. To explain these findings, we reasoned that Me-Indoxam and heparinase pre-treatment could enhance hGV binding to a different cell surface target on PMNs.

Two independent studies have shown that sPLA_2_s can activate human monocytes by binding to α_V_β_3_ and α_4_β_1_ integrins (3, 7), which are also expressed on PMNs (54, 55). hGIIA is structurally related to hGV (8, 56) and binds to α_V_β_3_ and α_4_β_1_ integrins through a specific domain, different from the catalytic center or the PLA_2_R1-binding site (3, 7). To investigate whether integrins could be involved in hGV-mediated release of VEGF-A_165b_, we stimulated PMNs with hGV in the presence of the two integrin antagonists P11 and TCS 2314, which inhibit α_V_β_3_ and α_4_β_1_, respectively (57, 58). The combination of these inhibitors markedly reduced the release of VEGF-A_165b_ (Figure 5E). The specificity of this finding is supported by the observation that the secretion of CXCL8/IL-8 was not affected (Figure 5F).

### Endogenous sPLA_2_s Modulate fMLF-Induced Neutrophil Activation in an Autocrine Fashion

**Figure 6:**
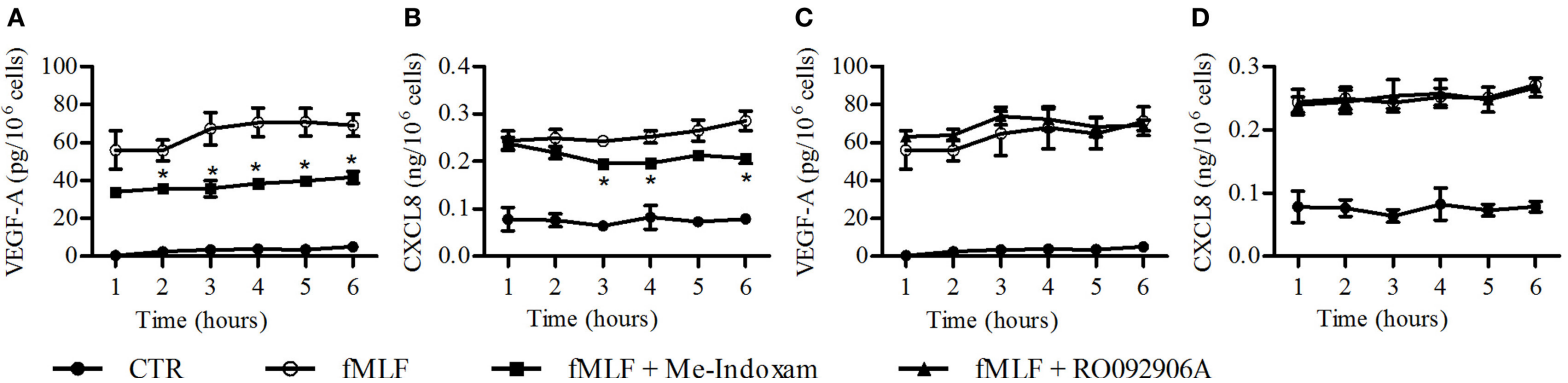
**Effect of Me-Indoxam (A,B) and RO092906A (C,D) on fMLF-induced vascular endothelial growth factor (VEGF)-A and CXCL8/IL-8 release from PMNs**. Cells were preincubated (37°C, 20 min) with or without Me-Indoxam and RO092906A (0.1 µM) and then stimulated (37°C, 1–6 h,) with fMLF (50 nM). VEGF-A **(A–C)** and CXCL8/IL-8 **(B–D)** release was determined by ELISA. Data are the mean ± SD of eight different preparations of PMNs. *p < 0.05 vs. fMLF alone.

fMLF induces the release of hGV by human PMNs (16, 19). We confirmed these results, showing an increased sPLA_2_ activity in supernatants from fMLF-stimulated PMNs which was inhibited by Me-Indoxam but not by the hGX-specific inhibitor RO092906A (Figure S2 in Supplementary Material) (59). Since we found that hGV induces VEGF-A, CXCL8/IL-8, Ang1, and VEGF-A_165b_ (Figure 3) release, it would be conceivable that also fMLF induced the release of these mediators in an hGV-dependent manner. However, in our model, fMLF only promoted the secretion of VEGF-A and CXCL8/IL-8 (Figure 4). Figure 3 shows that the concentration of hGV required for the release of VEGF-A (Figure 3A) and CXCL8/IL-8 (Figure 3B) was 0.3 µg/ml whereas that for the release of Ang1 (Figure 3C) and VEGF-A_165b_ (Figure 3D) was at least 1 µg/ml. Therefore, we measured the enzymatic activity of several concentrations of recombinant hGV and found that the sPLA_2_ activity in supernatants from fMLF-stimulated PMNs corresponded to a concentration of recombinant hGV ≤0.3 µg/ml (data not shown). Thus, it is likely that the release of hGV in response to fMLF is in our conditions insufficient to induce VEGF-A_165b_ and Ang1 release. Nevertheless, we asked whether fMLF-induced secretion of VEGF-A and CXCL8/IL-8 was mediated by the release of endogenous hGV. To address this question, PMNs were stimulated with fMLF in the presence or absence of Me-Indoxam or RO092906A. As expected, Me-indoxam reduced VEGF-A (Figure 6A) and CXCL8/IL-8 (Figure 6B) secretion while RO092906A had no effect (Figures 6C,D), suggesting an involvement of hGV but not hGX in fMLF-mediated neutrophil activation.

### Expression of hGV and Neutrophils in Lung Cancers

**Figure 7:**
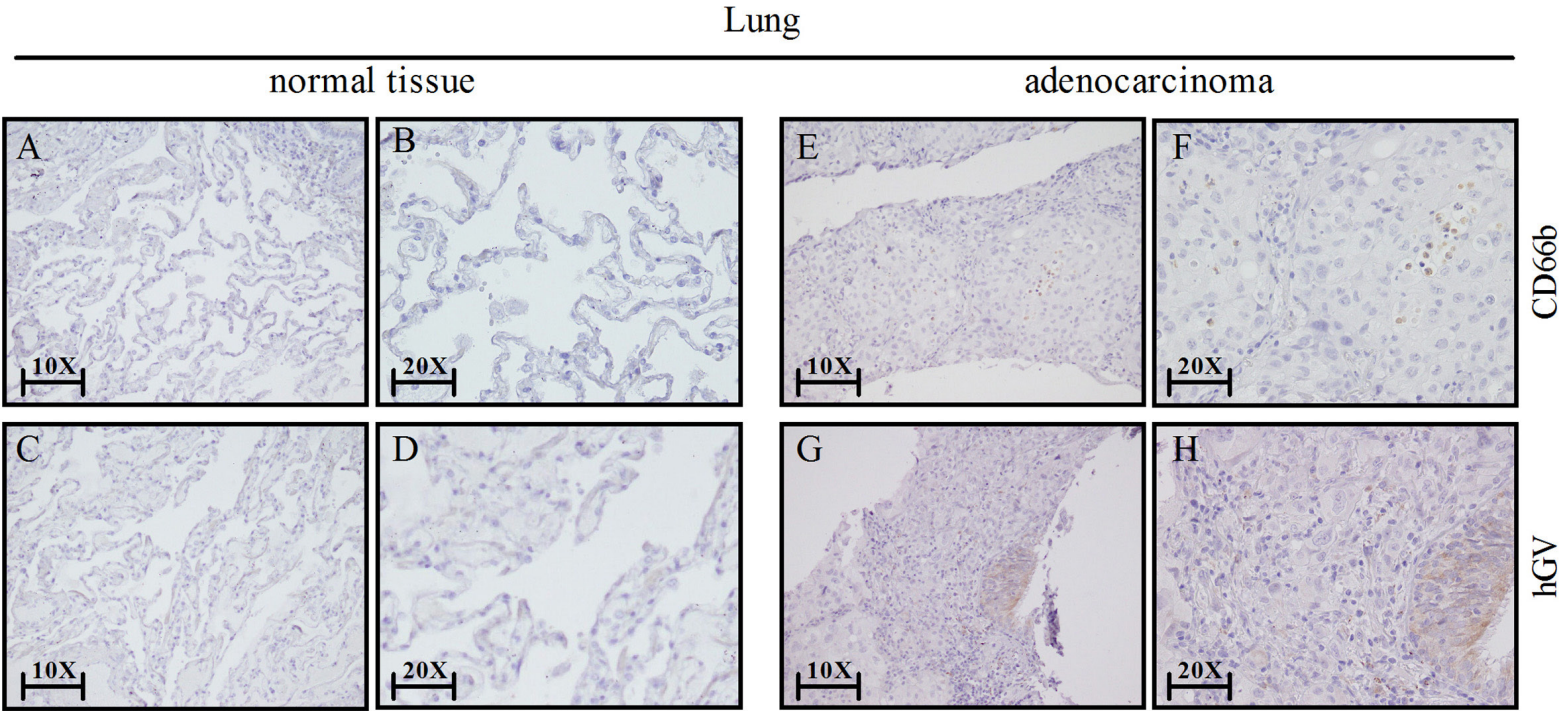
**Lung cancer express human group V (hGV) and neutrophils (CD66b^+^ cells)**. Sections of non-tumor **(A–D)** and tumor lung tissues **(E–H)** were stained for hGV [**(C,D,G,H)**; brown] or CD66b [**(A,B,E,F)**; brown]. Panels **(B,D,F,H)** display higher magnification (20×) of panels **(A,C,E,G)** (10×), respectively.

Our results show that hGV induces the release of proangiogenic and antiangiogenic factors by PMNs. Since angiogenesis is a hallmark of cancer-related inflammation (60) and PMNs infiltrate several human tumors (61), we assessed the expression of hGV and CD66b^+^ neutrophils by immunohistochemistry in neoplastic lung tissue samples. Figure 7 shows that hGV (Figures 7G,H) and CD66b (Figures 7E,F) were expressed in lung cancer samples but not in non-tumor areas (Figures 7A–D, respectively). These data suggest that hGV, expressed in lung adenocarcinoma microenvironment, can modulate tumor angiogenesis through the activation of TAN.

## Discussion

This study demonstrates that human PMNs constitutively express and contain several proangiogenic (VEGF-A_165_, VEGF-B, and Ang1) and antiangiogenic (VEGF-A_165b_) factors. Interestingly, human PMNs, similar to other circulating immune cells (e.g., basophils) (62), do not express lymphangiogenic factors (VEGF-C and VEGF-D). We observed that several human recombinant sPLA_2_s can selectively induce the release of pro- and antiangiogenic factors from PMNs. In particular, hGV and hGX, which are the most effective sPLA_2_s in activating human PMNs, stimulate the secretion of VEGF-A, Ang1, and CXCL8/IL-8, while hGV is unique in inducing VEGF-A_165b_ secretion. Critically and in contrast with VEGF-A, Ang1, and CXCL8/IL-8, the binding of hGV to integrins appears to be required for VEGF-A_165b_ secretion. Indeed, the effect of hGV on VEGF-A_165b_ secretion was abrogated by the addition of known inhibitors of α_V_β_3_ and α_4_β_1_ integrins, suggesting that hGV physically interacts with these integrins. Endogenous hGV is released by fMLF-stimulated human neutrophils and acts in an autocrine/paracrine fashion to modulate VEGF-A and CXCL8/IL-8 release. The translational relevance of these findings is supported by the increased expression of hGV in neutrophil-infiltrated lung cancer samples compared to non-tumor lung tissue.

On the one hand, neutrophils are modulators of inflammatory and tumor angiogenesis (36, 51). On the other hand, sPLA_2_s have been implicated in cancer (13, 15). In this study, we sought to investigate whether sPLA_2_s induce PMN secretion of angiogenic factors. Strikingly, we found a remarkable heterogeneity in the response to sPLA_2_s. hGV and hGX induced the highest levels of VEGF-A, Ang1, and CXCL8/IL-8. hGV and, to a lesser extent, hGIIA are the only tested sPLA_2_s to promote the secretion of the antiangiogenic factor VEGF-A_165b_. This molecule arises from an alternative splicing at the exon 8 distal site of *VEGF-A* mRNA and binds to both VEGFR-1 and VEGFR-2 (but not to co-receptor neuropilin-1) (42). However, VEGF-A_165b_ fails to induce VEGFR-2 tyrosine phosphorylation and to activate the downstream signaling pathway that characterizes the proangiogenic isoform VEGF-A_165a_ (42–44). As such, VEGF-A_165b_ restrains tumor growth and impairs angiogenesis in systemic sclerosis and peripheral artery disease (63–66). Our results support a model in which human neutrophil stimulation with hGV induces a mixed secretion profile whose functional outcome likely depends on the balance between proangiogenic and antiangiogenic factors. Further *in vitro* (e.g., endothelial cell tube formation assays) and *ex vivo* (e.g., immunohistochemistry analysis of neutrophil infiltration, hGV expression, and vascular patter in tumor biopsies) studies are required to define the role of hGV-induced neutrophil release of proangiogenic and antiangiogenic factors in inflammatory and tumor angiogenesis.

Human group V, an sPLA_2_ expressed by PMNs, induces VEGF-A, Ang1, and VEGF-A_165b_ secretion as early as 5 min after stimulation and independently of *de novo* protein synthesis. Indeed, freshly isolated PMNs contain these mediators as assessed in protein lysates. These results suggest that pro- and antiangiogenic factors are preformed and rapidly released upon activation. We also found that hGV stimulation does not increase *VEGF-A, Ang1*, and *VEGF-A_165b_* mRNA levels (data not shown), indicating that hGV preferentially acts by modulating the release of these factors rather than by acting at the level of transcription or alternative splicing. The signals responsible for the constitutive expression of angiogenic mediators, in particular VEGF-A_165b_, in PMNs are not known. VEGF-A is stored almost exclusively in the specific (β) granules (49), by contrast, Ang1 is predominantly located in the cytosolic fraction (50). Further studies are needed to understand the molecular details of VEGF-A_165b_ expression in resting and hGV-stimulated PMNs.

We also demonstrate that the hGV enzymatic activity and/or its binding to PLA_2_R1 as well as HSPGs are required for VEGF-A, Ang1, and CXCL8/IL-8 but not for VEGF-A_165b_ release that conversely requires the interaction of hGV with the integrins α_V_β_3_ and α_4_β_1_. The hGV-activated signaling pathways downstream of integrins required for VEGF-A_165b_ release have yet to be defined. fMLF induces the release of endogenous hGV that modulate the secretion of VEGF-A and CXCL8/IL-8 but not of VEGF-A_165b_, likely because of the low level of endogenous hGV, insufficient to drive VEGF-A_165b_ secretion. Nevertheless, we cannot exclude that in situations of high density of PMNs (e.g., in the context of acute inflammation), the concentrations of hGV may reach the minimum required to stimulate VEGF-A_165b_ release, possibly driving a switch toward antiangiogenic properties of PMNs.

To evaluate the *in vivo* relevance of our findings, we assessed the expression of hGV and CD66b (a marker of neutrophils) in human lung cancer and found a higher expression of hGV in neutrophil-infiltrated tumor lung tissue compared to non-tumor lung samples. These results suggest that PMNs may be activated by hGV in the context of lung cancer. Whether this interaction results in the release of proangiogenic and/or antiangiogenic factors has still to be determined. Since VEGF-A_165b_ restrains tumor growth and progression in several experimental models, our results support the exploration of the hGV–neutrophil axis in human lung cancer.

## Ethics Statement

The study protocol involving the use of human blood cells was approved by the Ethical Committee of the University of Naples Federico II, and written informed consent was obtained from blood donors undergoing thoracic surgery in according to the principles expressed in the Declaration of Helsinki.

## Author Contributions

Substantial contributions to the conception or design of the work; or the acquisition, analysis, or interpretation of data for the work; drafting the work or revising it critically for important intellectual content; final approval of the version to be published; and agreement to be accountable for all aspects of the work in ensuring that questions related to the accuracy or integrity of any part of the work are appropriately investigated and resolved: SL, FB, RI, AF, MG, VG, PE, GV, GL, MC, FG, and GM.

## Conflict of Interest Statement

The authors declare that the research was conducted in the absence of any commercial or financial relationships that could be construed as a potential conflict of interest.
