# Supplementary material for: Group V Secreted Phospholipase A2 Induces the Release of Proangiogenic and Antiangiogenic Factors by Human Neutrophils
Source: Front Immunol. 2017 Apr 19;8:443. doi: 10.3389/fimmu.2017.00443 (PMC5394767; doi:10.3389/fimmu.2017.00443)
Supplement: Supplementary file 1 [file image_1.pdf]

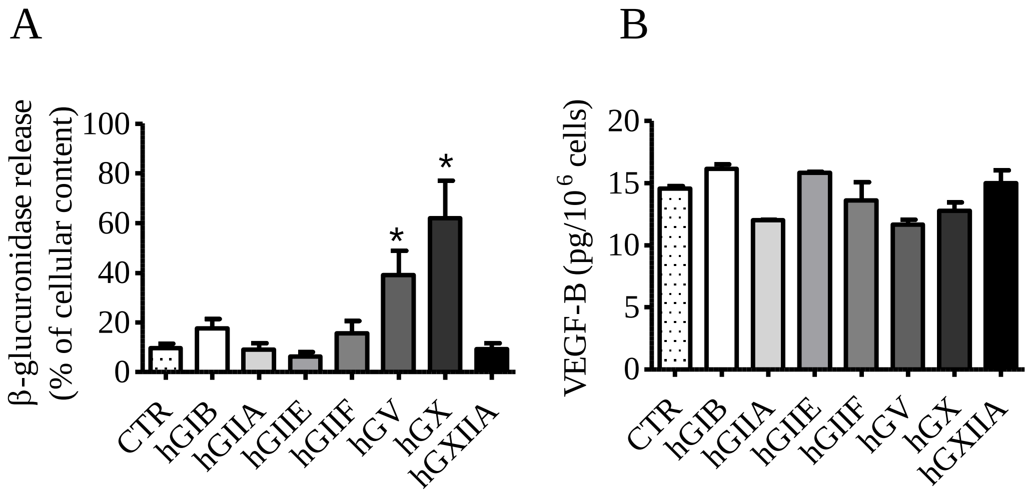

Supp Figure. 1. β-glucuronidase and VEGF-B release by $sPLA_2$-stimulated PMNs. PMNs were incubated (37°C, 3h) with $sPLA_2$ (5 µg/ml, hGIB, hGIIA, hGIIE, hGIIF, hGV, hGX, hGXIIA) or control medium **(A-B)**. At the end of incubation, the supernatants were collected and centrifuged (1000 x g, 4°C, 5 min). β-glucuronidase release **(A)** was determined by a colorimetric technique and expressed as a percentage of the total cellular content determined in cell aliquots lysed with 0.1% Triton X-100. VEGF-B **(B)** was determined by ELISA and the values are expressed as pg of VEGF-B per 10^6 cells. The results are the mean ± SD of six different preparations of PMNs.*****$p<0.05$ vs. control.
