# Supplementary material for: Group V Secreted Phospholipase A2 Induces the Release of Proangiogenic and Antiangiogenic Factors by Human Neutrophils
Source: Front Immunol. 2017 Apr 19;8:443. doi: 10.3389/fimmu.2017.00443 (PMC5394767; doi:10.3389/fimmu.2017.00443)
Supplement: Supplementary file 2 [file image_2.pdf]

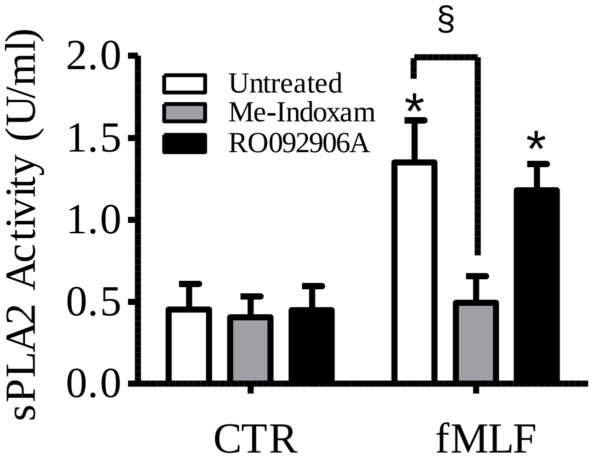

Supp. Figure 2. Effect of Me-Indoxam and RO092906A on fMLF-induced sPLA2 release from PMNs. The Cells were pre-incubated (37°C, 15 min) with or without Me-Indoxam and RO092906A (0.1 µM), and then stimulated (37°C, 10 min) with fMLF (50 nM). VEGF-A_{165b} **(A)** and CXCL8/IL-8 **(B)** release was determined by ELISA. Data are the mean ± SD of six different preparations of PMNs. **p*<0.05 *vs.* respective control. § p<0.05 *vs.* hGV alone.
