# Supplementary material for: Group V Secreted Phospholipase A2 Induces the Release of Proangiogenic and Antiangiogenic Factors by Human Neutrophils
Source: Front Immunol. 2017 Apr 19;8:443. doi: 10.3389/fimmu.2017.00443 (PMC5394767; doi:10.3389/fimmu.2017.00443)
Supplement: Supplementary file 3 [file table_1.pdf]

Supplementary Table 1 - Effects of Brefeldin A and Cycloheximide on the release of VEGF-A, CXCL8/IL-8, Ang1 and VEGF-A_{165b} from hGV-activated PMNs

	VEGF-A ($pg/10^6$ cells)	CXCL8/IL-8 ($ng/10^6$ cells)	Ang1 ($pg/10^6$ cells)	VEGF-A_{165b} ($pg/10^6$ cells)
Untreated	21.5±5.5	0.1±0.04	43.1±12.8	0.0±0.0
Brefeldin A	18.4±3.8	0.1±0.04	33.2±9.2	0.0±0.0
Cycloheximide	18.0±9.6	0.08±0.04	39.0±15.0	0.0±0.0
hGV	76.35±15.4	0.39±0.07	166.2±41.8	53.9±8.9
hGV +Brefeldin A	40.8±17.6*	0.24±0.08*	89.4±9.5*	28.2±2.9*
hGV + Cycloheximide	73.7±31.4	0.31±0.05	178.9±44.0	55.7±6.9

PMNs were preincubated for 30 min at 37 °C with medium alone (Untreated) or Brefeldin A (10 μg/ml) or Cycloheximide (10 μg/ml) and then were stimulated with hGV (3 μg/ml). At the end of incubation the concentrations of VEGF-A, CXCL8/IL-8, Ang1 and VEGF-A_{165b} were evaluated in the supernatants by ELISA.

Data are the mean ± SD of four experiments.

* p< 0.05 *vs.* hGV alone
