# Supplementary material for: Group V Secreted Phospholipase A2 Induces the Release of Proangiogenic and Antiangiogenic Factors by Human Neutrophils
Source: Front Immunol. 2017 Apr 19;8:443. doi: 10.3389/fimmu.2017.00443 (PMC5394767; doi:10.3389/fimmu.2017.00443)
Supplement: Supplementary file 4 [file table_2.pdf]

Supplementary Table 2 - Total level (supernatants plus cellular lysates) of VEGF-A, Ang1 and VEGF-A_{165b} in hGV-activated PMNs

	VEGF-A (pg/10^6 cells)	Ang1 (pg/10^6 cells)	VEGF-A_{165b} (pg/10^6 cells)
Untreated	242.6±34.6	283.5±61.4	261.5±71.5
hGV	273.4±25.4	354.7±55.8	297.7±98.9

PMNs were stimulated for 3 hours at 37 °C with medium alone (Untreated) or with hGV (3 µg/ml). At the end of incubation the concentrations of VEGF-A, CXCL8/IL-8, Ang1 and VEGF-A_{165b} were evaluated in the supernatants and in cellular lysates by ELISA.

Data are the mean ± SD of four experiments.
